# Delivering enhanced cardiovascular (Hypertension) disease care through private health facilities in Pakistan

**DOI:** 10.1186/1471-2261-13-76

**Published:** 2013-09-25

**Authors:** Muhammad Amir Khan, Wajiha Javed, Maqsood Ahmed, John Walley, Haroon Jahangir Khan

**Affiliations:** 1 Association for Social Development, Islamabad, Pakistan; 2 Nuffield Centre for International Health and Development, Leeds Institute of Health Sciences, University of Leeds, Leeds, UK; 3 Focal Person NCD, Directorate General of Health Services Punjab, Punjab, Pakistan

**Keywords:** Cardiovascular disease, Public private mix, Urban health

## Abstract

**Background:**

Cardiovascular diseases (CVDs) are one of the leading causes of death and disability in the world. Over 80% of CVD deaths take place in low-and middle-income countries. One-third of the population aged above 40 years suffers from Hypertension (HTN) and this is largely unreported as there is no registry for CVDs. No guidelines are available for use in health care facilities, especially private health facilities where practice among GPs varies considerably. We aim to conduct a Cluster Randomized Controlled trial delivering a quality HTN-CVD care package at strengthened private health facilities as compared to current practice at private health facilities.

**Methods/Design:**

A pragmatic cluster randomized trial, with qualitative and economic studies, will be conducted in Sargodha district of Punjab, Pakistan, from January 2012 to December 2016. At least 912 hypertensives will be registered in the two arms, six clusters per arm. The proposed cluster randomized controlled trial will evaluate the effects of delivering quality HTN-CVD care, through enabled private health care facilities, to achieve better case registration, adherence and hypertension control also blood glucose and serum cholesterol control. The trial will be conducted through the doctors and paramedics at private health facilities. Main outcomes are mean difference in Systolic blood pressure among the two arms. Secondary outcomes are mean change in total serum cholesterol levels and mean change in glycaemic control achieved in the adult hypertensive patients. Individual and Cluster level analysis will be done according to intention-to-treat.

**Discussion:**

Due to the high burden of disease where 1 in 3 individuals aged above 45 suffers from hypertension, topped with the fact that there is a dearth of a set of available, standardised guidelines for management, the disease is constantly on a hike in Pakistan. The government has made no effort to issue a set of guidelines adapted specifically for our population and this becomes more of a problem when managing CVD in urban population through private practitioners whose practices vary widely.If our set of context sensitive guidelines show an effectiveness in the proposed intervention districts it will be replicated in other such settings.

**Trial registration:**

Current Controlled Trials ISRCTN34381594

## Background

### Burden of CVD in Pakistan

The WHO report on Global atlas on cardiovascular disease prevention and control states that cardiovascular diseases (CVDs) are the leading causes of death and disability in the world. Over 80% of CVD deaths take place in low-and middle-income countries [1]. Despite convincing evidence that lowering of blood pressure decreases cardiovascular morbidity and mortality, the control of hypertension has been poor in developing countries due to a multitude of reasons.

According to National Health Survey Pakistan (NHSP) hypertension was shown to affect 18% of adults aged >15 years and around 33% of adults above 45 years [2]. In Pakistan there is a lack of adequate data on the CVD disease burden as well as the risk factors involved.

### Urban health and facilities available

A study done in urban settings (by Jafari et al.) found that 27.6%and 39.3% of hypertensive patients suffer from concomitant diabetes and hypercholesterolemia respectively, whereas 45.3% are smokers [3].

In urban areas, the availability and quality of the first level care public facilities is not adequate. This makes private clinics/ small hospitals an alternate “choice” for the first level care in urban areas. However, in the absence of a regulatory and/or partnership mechanism, the care delivery practices vary widely.

### Public–private mix models in Pakistan

In Pakistan two main models of public-private mix (PPM) emerged so far are the “social franchise” and the “district-led partnership” models. The variants of social franchise model (e.g. greenstar, MSS model) have been tested and found workable for delivering reproductive health and communicable disease care interventions in selected urban areas.

The district-led partnership model has been developed through an extensive and systematic exercise in Pakistan. The exercise included: a) strategic framework and intervention design; b) guidelines and products for planning, implementing and monitoring partnership interventions; c) piloting and evaluation of guidelines and products; and d) scaling-up of the refined model^a^ for TB, malaria, and more recently MDR-TB interventions.

### Involvement of the association

The Association is a not for profit company with vast experience of working in TB, lung health, reproductive health and cardiovascular diseases. The Association and Nuffield Institute have been the lead program technical partners for developing the district-led partnership model in Pakistan. Currently, the Association is responsible for implementing public-private partnership interventions in: 18 districts for DOTS and MDR-TB (TGF R-9), and 2 districts for RBM (TGF Round 7 and 10). This valuable experience and strong field presence will help the Association to implement the proposed CVD-HTN intervention in 1-2 of the districts with an ongoing public-private partnership for communicable disease control.

### Rationale

The proposed implementation research is to develop and evaluate a model of delivering CVD-HTN care package through a network of private clinics, already engaged in communicable disease control activities. The care delivery dimensions that need further attention include: chronicity of disease requiring relatively long-term liaison between patient and care provider, higher prevalence, combination of drugs and lifestyle interventions, and poorly defined management support arrangements at district level.

The CVD-HTN care delivery modalities and products are required for ensuring quality and planning expansion. The proposed exercise is to develop and evaluate an intervention for delivering quality CVD-HTN care through enabled private health facilities. The set of implementation products may include: case management desk guide, training module, recording/reporting and monitoring instruments, and communication tool. These products will cover the technical and operational aspects of managing cases i.e. screening, diagnosis, prescription, education, follow-up etc.

### Aim

The aim is to enhance the delivery of quality CVD-HTN care through private health facilities in urban settings. The conditions to be covered in the care package include: hypertension, type 2 diabetes, and hypercholesteremia.

### Primary objective

To compare the mean change in systolic blood pressure (10 mmHg) in adult patients (>25 years) receiving enhanced CVD-HTN care package with the mean change in systolic blood pressure (5 mmHg) for those receiving routine care at the private facilities in Punjab.

### Secondary objectives

•To compare the mean difference in systolic blood pressure (at least 6 mmHg) due to the intervention.

•To compare the mean change in total serum cholesterol (at least 4 mg/dl decrease) achieved in the adult hypertensive patients.

•To compare the mean change in glycaemic control in the adult type 2 diabetes patients (among registered hypertensive cases) receiving enhanced CVD-HTN care package (2%change in HbA1c) in comparison with the mean change for those receiving routine CVD-HTN care at the private health facilities(1%change in HbA1c).

•To compare the tobacco cessation rate in intervention arm (41%) and control arm (8%).

•Record adverse events of Cardiovascular disease (MI, angina) and cerebro-vascular disease (stroke) among the two arms of the study.

•To conduct incremental cost effectiveness analysis of managing adult hypertensive patients with or without associated conditions (Type 2 diabetes and hypercholesterolemia) at private care facilities in Punjab, Pakistan.

### Research question

•How effective and feasible is to deliver and manage quality care for the control of selected cardiovascular diseases (i.e. hypertension, type-II diabetes, and hyper-cholesteremia) in adults (i.e. > 25 years), through enabled private health facilities as compared to current practice, in urban settings?

The control of selected cardiovascular diseases will be assessed by measuring change in blood pressure, blood glucose, and serum cholesterol.

### Hypothesis

•The enabling of private health facilities for cardiovascular disease intervention for adult hypertensive patients will improve the control of hypertension by decreasing mean systolic BP by at least 6 mm Hg.

•The enabling of private health facilities for cardiovascular disease intervention for adult hypertensive patients will improve the control of Blood glucose (HbA1C < 7.5%) and serum cholesterol (<200 mg/dl) by at least 15% (as compared to the current practice).

## Methods/Design

### Study design

A pragmatic parallel arms cluster randomized controlled trial (c RCT) with two arms will be conducted from January 2012 to December 2016 with a duration of 60 months.

**Figure 1:**
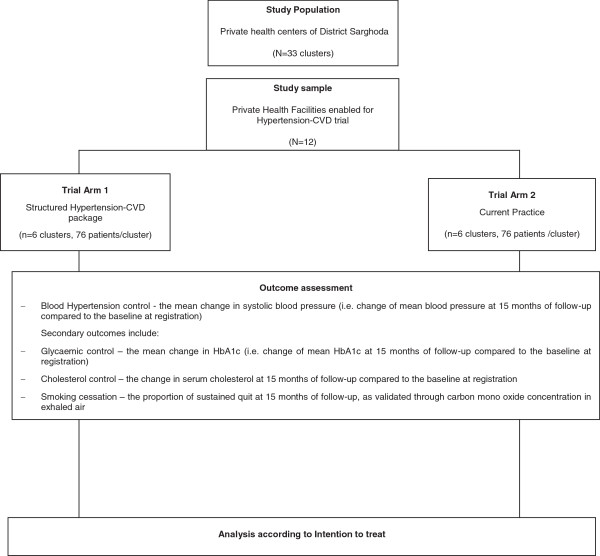
Flow chart of study.

The proposed cluster randomized controlled trial will evaluate the effects of delivering quality HTN-CVD care, through enabled private health care facilities, to achieve better adherence and hypertension control also blood glucose and serum cholesterol control. The trial will have two arms (Figure 1).

### Interventions

#### Intervention arm

Quality care will be delivered to adult hypertension patients, through enabled private healthcare facilities. The inputs for delivering quality care at enabled facilities will include: a) context sensitive operational guidelines^b^, b) supplement equipment and communication, c) training and supervisory support, d) patient education materials. The care delivery dimensions to be addressed will include: a) enhanced screening and diagnosis, b) standardized prescription, c) interactive education of patient and family, d) follow-up and adherence, e) recording, reporting and monitoring, f) referral linkage with respective district hospital, g) district health office engagement and support, and h) technical support for in-country research uptake.

#### Control arm

The control for comparison is the current care practices (for hypertension and associated conditions) at the private health care facilities. The only addition will be a) enhanced screening and diagnosis, b) introduction of HTN-CVD register for collecting core data set on patients attending these control facilities. In ‘current practice’, each practitioner manages HTN-CVD cases, as per his/her self-determined regimen and without reference to any specific guidelines and/or tools.

The health staff at a facility will offer the same care package to all its HTN-CVD cases, regardless of their social strata and other preferences. This will reduce the chances of intra-cluster contamination as well as strengthen and simplify the implementation of prospective evaluation. All trial patients will be asked to arrange their prescribed drugs, so that the trial reflects the real life practice in private health care delivery system.

### Study setting

Sargodha is the tentatively proposed district for implementing the research. About one-third of the district population lives in urban areas (i.e. 1 million out of 3.1 million). About 38% of the urban population is above 25 years of age (i.e. 380,000), and about a quarter of these are expected to be hypertensives (95,000).

In Sargodha, the Association has actively been engaged in supporting the implementation of various disease control interventions through district health systems. These include communicable diseases like TB-DOTS and sexually transmitted infection (STI) control, tobacco cessation, public-private partnership, advocacy-communication and social mobilization for disease control, drug management enhancement etc. This experience-based understanding and trust with the district health office would help partners to implement the proposed public-private partnership innovation.

### Study population

In these two districts, *12* private health facilities will be selected into the trial.

#### Identifying participants

A group of private facilities has already been selected for TB-PPM intervention in the district. These facilities have been selected on basis of objective functioning and willingness criteria. The private facilities for HTN-CVD trial will be identified from the already functioning facilities in the district.

The individuals attending the outpatients will be identified and screened for HTN and subsequently for type 2 diabetes, hypercholesterolemia and smoking, as per agreed care delivery guidelines.

#### Approaching participants

The eligible facilities will be approached through district health office for possible inclusion in the trial. Through respective health facility, communal consent will be arranged from the respective catchment populations. The facilities where both staff and the catchment population consent to participate will be selected in the trial.

#### Inclusion/exclusion criteria

Among these private facilities ,all newly diagnosed hypertensive (systolic blood pressure >140 mm Hg, diastolic blood pressure >90 mm Hg) male and female patients aged 25 years or more from the catchment population of the respective facility will be included, after an informed consent. The patients to be excluded from the trial are: a) pregnant women, b) persons with advanced chronic disease, c) person with conditions that cause secondary hypertension, d) person with known history of hypertension and/or CVD treatment in the past, e) person not likely to stay in the area for the required follow-up period of 15 months.

Patients who are pregnant and have hypertension will be referred to a gynecologist, patients with advanced chronic disease to a general medical practitioner, and those with a known history of hypertension and/or CVD treatment in the past will be referred to a cardiologist. Only patients who are new cases of hypertension are included in the trial. People with known hypertension are excluded from the trial as they would already be on some sort of antihypertensive drug regimen which may mask the effects of our intervention.

#### Recruiting participants

The eligible for inclusion patients will be informed and offered to participate (see inclusion/ exclusion criteria above). The patient is recruited only if he/she consents to participate.

Once recruited, each patient in the:

– control arm will be offered standardized diagnosis and care as per current practice, whereas intervention arm will be offered an enhanced HTN-CVD care including standardized diagnosis, prescription, follow-up, and monitoring.

– intervention arm will also be educated on life style modifications recommended for the individual, whereas control arm patients will receive information brochure.

– both arms will be asked to arrange the prescribed medicine through their own sources. However, access to baseline BP, HbA1c, cholesterol examination will be facilitated, for both arms, through trial resources.

Both arms will be requested to adhere to the treatment protocols.

### Data management procedures

Most of the data will be collected as a part of the care delivery process by doctors and paramedics on the ‘CVD card’. Other data related especially to trial will be recorded on the ‘trial registration form (TRF). Research assistant (RA) will visit the identified Lab on monthly basis and will collect test results. RA will visit each centre on monthly basis and will i) check each TRF for completeness and correctness, and those found with missing data will be acted upon accordingly. ii) correlate the Lab results with entries on TRF iii) check for drugs and supplies and reimburse for gaps. RA will send a copy of TRF and Lab test results to the central office i) at test one i.e. baseline ii) at test two i.e. final outcome, for data entry, cleaning and analysis.

#### Data confidentiality and data entry

Each form will be sent as mentioned above by a registered courier service in a sealed envelope. The forms will be kept under lock and key in the central ASD office with only the PI and project coordinator having access to them. Data entry will be done twice by two independent data entry operators to minimize typing errors. Data will be entered on SPSS 20.

### Statistical considerations

#### Sample size

A total of twelve clusters^c^ (i.e. six clusters in each trial arm) will be included in the study, with a minimum of 76 patients recruitment in each cluster and a total of 912 patients in the trial. The assumptions are: a) an intra-class correlation coefficient (ICC) of 0.02, b) design effect of 2.5 [4], c) and standard deviation of blood pressure as 11 mm [5] d) lost to follow-up of 25%. This sample size will give 94% power using 5% level of significance for detecting an assumed difference in the mean change of systolic blood pressure of 6 mm Hg between the intervention and the control arm [6,7].

For a sub group analysis of hypertensive patients with concomitant type-II diabetes, the main assumption is that 27.6% of the recruited hypertensive will have type-II diabetes i.e. 21 patients out of 76 hypertensive recruited at each clinic will be have diabetes [3]. The proposed sample size will give 86% power using 5% level of significance for detecting an assumed difference in mean change of HbA1c of 1% [8,9] between the intervention and the control arms. The assumed standard deviation of HbA1c is 2% [10], and intra-cluster correlation is 0.02 [6,7].

For a sub group analysis of hypertensive patients with concomitant hyper-cholesteremia, the main assumption is that 39.3% of the recruited hypertensive will have hyper-cholesteremia i.e. 30 patients out of 76 hypertensive recruited at each clinic will be have hyper-cholesteremia [3]. The proposed sample size will give 100% power using 5% level of significance for detecting an assumed difference of 4.9 mg/dl [11] between the group means. The assumed standard deviation is 2.9 mg/dl [11], and intra-cluster correlation is 0.02 [6,7].

For a sub group analysis those hypertensive patients who smoke, the main assumption is that 34.5% of the recruited hypertensive will be tobacco smokers i.e. 27 out of 76 hypertensive recruited at each clinic will be smokers [3]. The proposed sample size will give 100% power using 5% significance level for detecting a difference between the group proportions of 0.33 when the intra-cluster correlation is 0.02 [6,7].

#### Planned analysis

The results in each cluster would be analyzed on the basis of “intention to treat”. This means the quality of care and service utilization outcomes of each patient will be counted in the intervention for the cluster, regardless of their actually accepting or availing the offered services.

#### Planned statistical tests

Cluster and individual level analysis will be done on SPPS version 17. Histograms will be made for the continuous variables e.g. age, systolic blood pressure, diastolic blood pressure, fasting blood glucose, HbA1c etc.

Descriptive statistics will be computed: proportion for categorical variables, mean and standard deviation for continuous variables having normal distribution, median and inter quartile range for continuous variables having skewed distribution.

a. Difference of mean systolic blood pressure at baseline and then at 15 months of follow up between the intervention and the control arm will be measured by independent t-test. Difference of mean systolic blood pressure at baseline and at 15 months of follow up in the intervention arm/control arm will be measured by the paired sample t-test. Mean change of systolic blood pressure between intervention and the control arm will be measured by t-test. P-value of < 0.05 will be considered significant.

b. Difference of mean HBA1c at baseline and then at 15 months of follow up between the intervention and the control arm will be measured by independent t-test. Difference of mean HBA1c at baseline and at 15 months of follow up in the intervention arm/control arm will be measured by the paired sample t-test. Mean change of HBA1c between intervention and the control arm will be measured by t-test. P-value of < 0.05 will be considered significant.

c. Difference of mean total cholesterol levels at baseline and then at 15 months of follow up between the intervention and the control arm will be measured by independent t-test. Difference of mean total cholesterol levels at baseline and at 15 months of follow up in the intervention arm/control arm will be measured by the paired sample t-test. Mean change of total cholesterol levels between intervention and the control arm will be measured by t-test. P-value of < 0.05 will be considered significant.

d. Proportions of adult Type 2 diabetes patients having htn control (<140/90) in the two arms will be compared by conducting chi-square test. P-value of < 0.05 will be considered significant.

e. Residual confounding will be taken care of by multivariable regression (MLR)analysis. Strartified MLR will be done with the different groups of medications being as different strata eg, Beta blockers, ACE inhibitors, ARBs, Calcium channel blockers etc. being as different strata. Results will be presented in a stratified analysis. This will take care of confounding my different effects on BP from different groups of anti hypertensive drugs.

f. Incremental cost and effect will be calculated for the intervention and the control arms and subsequently cost: effect ratio will be calculated.

### Phases of the trial

**Figure 2:**
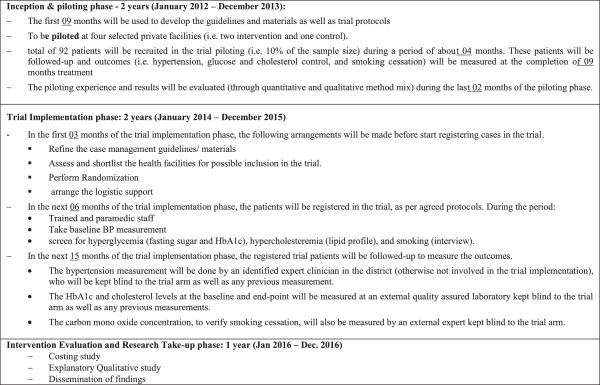
Phases of the trial.

The trial will have the following three phases: Refer to Figure 2.

1) Inception & piloting phase. The main purpose of the two-year phase is to develop, pilot and refine the care delivery guidelines/ materials and trial design/ protocols before embarking upon the trial registration.

– The piloting experiences and results will enable the research team to:

– Assess the feasibility and acceptability, and then accordingly modifythe process for health facility selection (i.e. facility assessment, providers’ and communal consent for the trial etc.) and management support (logistic supplement, reporting, monitoring etc.)

– Assess the feasibility and acceptability, and then accordingly modifythe case management guidelines i.e. diagnosis, treatment, record keeping, follow-up etc.

– Assess the feasibility and robustness of the research protocols, then accordingly modify, the protocols for case inclusion/ exclusion, recruitment rate, and periodic assessment (research related).

– Revisit the assumed proportion of diabetics, dyslipidemic and smoker patients among adult hypertensive patients in local context.

– Revisit the assumptions about sample size calculation to meet the power of the study such as mean change (± standard deviation) in systolic blood pressure, glycaemic and cholesterol control, and tobacco cessation between intervention and control arms.

– Assess and modify, if needed, the proposed statistical methods to analyze the trial data.

– The qualitative interviews with providers (± patients) for assessing the feasibility during the trial piloting will also be adapted subsequently for the trial assessment. In short the trial piloting will inform the decision to implement the trial to get optimal results and valid evidence.

2) Trial Implementation phase

– In the first 03 months of the trial implementation phase, the following arrangements will be made before start registering cases in the trial.

– Refine the case management guidelines/ materials Assess and shortlist the health facilities for possible inclusion in the trial. Randomization :The ASD central trial unit, under the supervision of Trial Steering Committee, will then randomize the facilities into two trial arms. Randomization preformed will be simple randomization. Each participating facility (cluster) will be given a unique ID which the investigators will be blinded to.12 different sheets of paper will be taken and the unique ID of each cluster will be written on the paper, which will be folded and sealed into an envelop.12 such sealed envelopes will be made. Then by a simple procedure, 6 sealed envelopes will be selected by hand picking them randomly from the pile of 12 sealed envelopes.6 will be hand picked for the intervention arm and 6 sealed envelopes for the control arm. The Steering committee will consist of a director, two people from ASD and two people from in-country (PMRC) and international partners (WHO).

– At least two doctors and two paramedics from each participating facility will be trained. The intervention facility staff will be trained on enhanced case management guidelines and materials including recording reporting and enhanced follow-up and referral, whereas the control facility staff will be trained only to register and report, use patient education brochure, and access HbA1c and cholesterol testing for trial patients.

– In the next 06 months of the trial implementation phase, the patients will be registered and enrolled in the trial, as per agreed protocols. During the period the baseline hypertension measurement will be validated by an external expert examination, kept blind to the trial arm as well as any previous measurements.

– The case management enhancement in the intervention arm will include: case management desk-guide for patient care, flip chart and brochure for patient education, mobile link for late patient retrieval, referral for advanced care (if needed), regular monitoring and support.

– Once the required number of trial patients is enrolled in the trial, the subsequent patients will receive HTN-CVD and associated care from the respective facility (as per prevailing case management protocols i.e. either intervention or control) but their results will not be included in the trial evaluation.

– The research team will closely monitor the inclusion and exclusion of trial patients, mainly to identify and respond to any deviation from the research protocols and/or case management processes.

– In the next 15 months of the trial implementation phase, the registered trial patients will be followed-up to measure the outcomes.

– All patients will pay for the case management services, as per facility protocols. However, the measurement of hypertension, HbA1c, serum cholesterol and carbon mono oxide in exhaled air will be paid from the project sources (as these measurements are being made solely for the trial evaluation). The referral to district headquarter hospital, as required, for specialist consultation (e.g. cardiology, ophthalmic examination, renal assessment, drug side effect) will be managed as per agreed guidelines for intervention arm and as per current routine in the control arm. The specialist facility will be encouraged to refer back patients to the respective referring facility, after providing the requested specialist care.

– The case management record on each patient will be maintained in the chronic disease register (to be developed and introduced through the trial inputs). The chronic disease register at each facility will provide the data for detailed analysis on case management experiences in the two trial arms.

– The modalities for periodically monitoring the participating facilities (including reviewing records and collecting reports) will be developed during the initial development phase (see section 12 below). The research team will ensure that each participating facility is monitored as per guidelines agreed for the respective facility (i.e. intervention and control).

– The research team will also supplement the logistic inputs at the participating facilities. This includes maintenance of BP apparatus, glucometer and supplies, print materials etc. at the participating facilities.

3) Intervention Evaluation and Research Take-up phase

#### Costing study

A costing analysis will be designed and conducted to inform programmatic decisions by managers and policy makers for possible scaling-up in other districts with ongoing PPM for communicable disease control. The minimal is the incremental cost effectiveness ratio (ICER) analysis of the provider cost (i.e. health department and private facility). The ICER will focus mainly on cost of additional inputs (training, materials, monitoring etc.) at private facility level to achieve the outcomes. This analysis is more relevant for the health department decision makers to consider scaling-up the intervention. The main source of cost data will be records and key informant interviews. We will also explore the possibility of covering, to an extent possible, the patient perspective to the care for HTN and associated conditions. This might involve surveying the registered patients for costing and coping experiences.

#### Explanatory qualitative study

Qualitative interviews with selected care providers (6), and patients (12)^d^ will help to better understand the experiences. A qualified anthropologist, with help of other members, will develop the interview schedules and conduct the semi-structured interviews with various individual categories of interest. The study would collect data on a range of social, economic, service access and quality etc. factors from care providers as well as patients and their families to assess the social and administrative feasibility of said interventions in the programme context and inform refinements before scaling-up to other parts of the country.

#### Dissemination

The results/experiences and products of research will also be shared with wider stakeholders through: a) existing partner networks (e.g. COMDIS,), b) presentations at national and international conferences (e.g. White Ribbon Alliance, World Federation of Public Health Associations), and c) access to resource centers/websites more widely used e.g. WHO.

The scientific papers, based on results of the trial and associated studies, will be submitted to international and national peer-reviewed journals. The potential journals to target includes: BMC cardiovascular disease, Lancet, British Medical Journal, WHO Bulletin, BMC Implementation Science-process evaluation study(s); Health Policy and Planning, Reproductive Health Matters-social science (qualitative studies).

## Discussion

### Current situation

Due to the high burden of disease where 1 in 3 individuals aged above 45 suffers from hypertension, topped with the fact that there is a dearth of a set of available, standardised guidelines for management, the disease is constantly on a hike in Pakistan. The government has made no effort to issue a set of guidelines adapted specifically for our population and this becomes more of a problem when managing CVD in urban population through private practitioners whose practices vary widely. If our set of context sensitive guidelines show an effectiveness in the proposed intervention districts, the 6 facilities in control arm will also be enabled for implementing the enhanced HTN-CVD care guidelines and materials. The enabling primarily will include staff training, guidelines and materials and monitoring support. Consultation with selected provincial health department staff will be conducted for their considering and planning (if possible) the scaling-up of HTN-CVD PPM intervention in other 31 districts of Punjab where PPM for communicable disease (TB) control is already being implemented through GFATM grant.

### Addressing bias, error and limitations

Comparing the socio-demographic and health services profiles of the private facilities does not indicate any significant difference that can potentially lead to differences in care seeking behavior and/or quality of service. The proposed cluster randomization is expected to address the potential selection bias across the two trial arms.

The process of equal screening and diagnosing training in both arms will lead to similar disease intensity patients being recruited in both arms. Although similar training of health professionals in both arms in regards to better screening this will eliminate selection bias, it will lead to a inability to measure ‘case detection rate’ due to better screening.

The lack of masking (blinding) could introduce bias through two routes i.e. “incorrect measurement” and “leakage” from one group to another. There was a possibility of incorrect measurement of “blood pressure outcomes” by the facility doctor, who is aware of the intervention allocation. This possibility of measurement bias will be reduced through an unrelated external BP monitoring doctor who is unaware of the intervention allocation. Contamination across clusters is prevented by keeping a minimum distance between clusters.

The proposed randomization does not allow asking and responding to individual patient preferences. So the trial will not answer the question what would happen if health services are able to offer intervention according to individually tailored patient needs/ convenience. Further work will be needed to test the approaches sensitive to individual preferences, in the local programme setting.

The proposed method of randomization, ie concealed simple randomization has its own limitations. This method does not address the balance of baseline covariates. An alternative approach could have been addresses the need to control and balance the influence of covariates by using stratified randomization. This method can be used to achieve balance among groups in terms of baseline characteristics (covariates) of health facilities based on socio demographics of catchment population they feed into, size of health facility, patient load etc. Different strata /blocks of health facilities may have been made and then simple randomization done in each strata to achieve a balance of covarites. This could have minimized the biased that simple randomization does not address.

### Ethical considerations

The proposed study design, i.e. a randomized controlled trial, is considered best for a prospective comparison of two or more options or interventions. The relevant details (e.g. selection of study and control population, sampling technique and sample size, statistical methods etc.) have carefully been considered to ensure valid measurements.

The endorsement of EDO (H), after explaining the trial significance and methods, will be obtained. Then a survey of eligible private health facilities will form the basis for selection of well functioning and willing to participate health facilities (i.e. consent to participate in the trial). At each selected health facility communal consent (from the respective catchment population) will be obtained, by inviting an agreed group of key community leaders. The facilities where communities consent to participate will then be randomized into two trial arms. The proposed process for seeking consent from the private providers and respective communities is considered ethical for the proposed cluster randomised controlled trial.

The selection of current practice as a control arm is also considered ethically acceptable. As there is no existing evidence for an association between the proposed interventions and better outcomes, the cluster randomization into trial arms is considered justified. No participant will be subjected to an additional burden or a potentially coercive influence, or deprived of any care that he/she would ordinarily receive. The outcome measurement will be based on an objective and quality assured data, gathered during the care delivery process. There are no apparent issues related with confidentiality of information about individual client in the trial.

The University of Leeds Research Ethics Committee, granted the trial ethical approval on 20 March 2012, ref: HSLTLM11026. National Bioethics Committee (NBC) Pakistan has granted ethical approval for the proposed project on 30 April 2012, ref: NBC:89 The trial has also been registered with the Current Controlled Trials ISRCTN34381594.

## Endnotes

^a^GFATM Rounds 7 and 10 for malaria, and Rounds 6, 8 and 9 for TB and MDR-TB care interventions.

^b^Operational guidelines, training materials and communication materials will be developed to deliver care for the selected non-communicable conditions. Guidelines in draft available, Public-private partnership in Cardiovascular Disease & Hypertension, cited from http://asd.com.pk/CVD%20Guidelines/CVD%20deskguide%20(intervention%20arm).pdf.

^c^A cluster is a private health care facility with its catchment population.

^d^6men–3 un/successful from urban localities, and same for women.

## Competing interests

The authors declare that they have no competing interest (financial or otherwise) in this publication.

## Authors’ contributions

MAK carried out the background literature review, identified the research gap, conceived the research question and objectives and proposed the trial. MA and WJ contributed towards study methodology, manuscript writing and its critical review for intellectual content. WJ performed the sample size estimation and planned statistical analysis. JW reviewed the study design with regards to the health systems component and provided technical inputs for the overall project. HJ helped in designing the trial in NCD Pakistan programme context and gave technical guidance for tool development in the intervention arms. All the authors read and approved the final manuscript.

## Pre-publication history

The pre-publication history for this paper can be accessed here:

http://www.biomedcentral.com/1471-2261/13/76/prepub
